# Cinematographic continuity edits across shot scales and camera angles: an ERP analysis

**DOI:** 10.3389/fnins.2023.1173704

**Published:** 2023-07-14

**Authors:** Javier Sanz-Aznar, Luis Emilio Bruni, Salvador Soto-Faraco

**Affiliations:** ^1^Section of Communication, Department of Hispanic Studies, Literary Theory and Communication, University of Barcelona, Barcelona, Spain; ^2^Augmented Cognition Lab, Section for Media Technology, Department of Architecture, Design and Media Technology, The Technical Faculty of IT and Design, Aalborg University, Copenhagen, Denmark; ^3^Multisensory Research Group, The Center for Brain and Cognition, Pompeu Fabra University, Barcelona, Spain; ^4^Institució Catalana de Recerca i Estudis Avançats (ICREA), Barcelona, Spain

**Keywords:** EEG, ERP, editing, cinema, neurocinematics, perception, film, continuity

## Abstract

Film editing has attracted great theoretical and practical interest since the beginnings of cinematography. In recent times, the neural correlates of visual transitions at edit cuts have been at the focus of attention in neurocinematics. Many Event Related Potential (ERP) studies studies have reported the consequences of cuts involving narrative discontinuities, and violations of standard montage rules. However, less is known about edits that are meant to induce continuity. Here, we addressed the neural correlates of continuity editing involving scale, and angle variations across the cut within the same scene, two of the most popular devices used for continuity editing. We recorded the electroencephalographic signal obtained from 20 viewers as they watched four different cinematographic excerpts to extract ERPs at edit points. First, we were able to reproduce the general time and scalp distribution of the typical ERPs to filmic cuts in prior studies. Second, we found significant ERP modulations triggered by scale changes (scale out, scale in, or maintaining the same scale). Edits involving an increase in scale (scale out) led to amplification of the ERP deflection, and scale reduction (scale in) led to decreases, compared to edits that kept scale across the cut. These modulations coincide with the time window of the N300 and N400 components and, according to previous findings, their amplitude has been associated with the likelihood of consciously detecting the edit. Third, we did not detect similar modulations as a function of angle variations across the cut. Based on these findings, we suggest that cuts involving reduction of scale are more likely to go unnoticed, than ones that scale out. This relationship between scale in/out and visibility is documented in film edition manuals. Specifically, in order to achieve fluidity in a scene, the edition is designed from the most opened shots to the most closed ones.

## 1. Introduction

In his 1900 film *As Seen Through a Telescope*, G. A. Smith cut from a medium-wide shot (the standard at the time) of a man looking through a telescope, to a close-up of a woman’s ankle. This had the effect of aligning the viewer’s experience with that of the character in a seamless, continuous flow; a powerful narrative device was developed. Cinematography involves the creation of a sense of flow and continuity from a succession of shots with different viewing angles, times, spatial locations and characters. This sense of flow is created in the brain of the viewer, but it is strongly influenced by editing. It is not surprising that throughout the history of cinema, film editing has been extensively studied and remains one of the most essential technical aspects of the medium (Shklovsky, 1928/1971; Mitry, 1963/2002; Deleuze, 1984; Cutting et al., 2011).

In recent decades, with the interest of cognitive neuroscience in the study of different artistic disciplines (e.g., Ramachandran and Hirstein, 1999; Hasson et al., 2008; Van de Cruys and Wagemans, 2011), film editing has once again been at the forefront regarding cinematographic research. Addressing film editing is relevant for the advance of film studies as well as for understanding cognitive processes and their neural underpinnings (Matusz et al., 2019; Soto-Faraco et al., 2019). For example, the study carried out by Silva and her research team analyzed shot changes across editing cuts to expand neuroscientific knowledge about the memorization and mental organization of episodic events (Baldassano et al., 2018; Silva et al., 2019), while from a different perspective, Smith developed a theory applicable to continuity in film editing grounded on current knowledge about cognitive processes (Smith, 2012).

Edit cuts may serve a wide variety of purposes as narrative, aesthetic and emotional devices in the context of cinematography. These techniques may pursue the creation of smooth visual continuity flow (continuity edits) or else breaks that flag important narrative boundaries (such as those spanning different scenes). Here, we are especially interested in how different types of shots are combined in continuity editing. This is an essential aspect in cinematographic construction and typically represents one of the most relevant subjects of cinematography handbooks (Reisz and Millar, 1971; Marimón, 2015), as continuity editing is designed to help direct the viewer’s attention toward the narrative of the film and away from the cinematographic technical artifact (Burch, 1969). Subverting these rules breaks continuity, and is sometimes used to expressively highlight shot changes, seeking to have an impact on the audience (Marimón, 2015). However, excessive use of forbidden editing [as it was called by Bazin, 1958/2004] can break the narrative virtuality of the film, diverting the viewer’s attention from the narrative content.

Specifically, we are interested in how different types of cuts for continuity affect the viewer depending on changes in shot scale and the filming angle. Cuts involving scale changes (scale-out: from a close to an open shot, or scale-in: from an open to a close shot) are typically used in cinematic language for managing the emotional tension of the scene. A typical filmic structure would start with a wide shot showing the context of the staging, and make the scene unfold dramatically by scaling-in to a closer shot focusing on the main character(s) and their emotional expressions (Marimón, 2015). Cuts involving angle variation (the point of view of the camera moves with respect to the object being filmed) are another typical device used in cinematic language, for example when filming a conversation, alternating specific shot angles for each character. In film theory, the angle variation is bounded by the 30-degree rule, whereby 30-degree changes are considered to be the minimal variation needed to maintain a sense of continuity in the viewer (Shimamura, 2013; Marimón, 2015).

The study of cinematographic editing in cognitive neuroscience has employed different methods including measurement of response speed and accuracy, eye gaze, and neurophysiological measures with functional magnetic resonance imaging (fMRI) and electroencephalography (EEG). For example, with regard to fMRI, Magliano and Zacks (2011) analyzed cuts depending on their continuity or discontinuity in space, time, and action, and discovered that spatial–temporal changes and action changes produced different neural patterns, compared to purely continuity edits. Based on eye-tracking studies, amongst other analytical approaches, Smith and Henderson (2008) and Smith (2012) proposed the *Attentional Theory of Cinematic Continuity* (AToCC). The AToCC is based on the viewer’s processing of the visual image in relation to gaze shifts and fixations, proposing how a sense of continuity is achieved across the shot change, and the editing techniques that favor it. For example, Smith proposes that attentional cues at the end of one shot may be used to produce a gaze shift in the viewer in order to make the cut to the next shot less noticeable. Regarding EEG approaches, the main lines of analysis cover the temporal and the frequency domains of neural responses. Heimann and her team (2017) combined both to investigate how the shot change affects neural activity when the 180-degree rule is broken. Their results showed that cuts, in general, elicit early event related potentials (ERPs) similar to those produced by syntactic violations in language and action sequences, and also suggested that the left–right reversal resulting when the 180-degree rule is broken caused an orienting deficit, reflected it in the event-related desynchronization/synchronization (ERD/ERS) pattern. We have recently reported a shot change study addressing ERD/ERS analysis, focusing on common patterns triggered by different types of continuity cuts (Sanz-Aznar et al., 2021). Our results showed a common pattern of ERD/ERS for continuity cuts related to theta and delta frequency bands, mainly in parietal electrodes. During the first 188 ms following an edit cut there is synchronization in theta rhythms and, between 250 and 750 ms after, a desynchronization in the delta frequency band.

In the present study we concentrate on the consequences of different types of continuity edit cuts on ERPs extracted from the EEG. ERP analysis provides a continuous measure of processing between a stimulus and a response with better temporal resolution than other physiological measures, making it possible to determine with precision the stage or stages of processing that are affected by a specific experimental manipulation (Luck, 2014). This possibility is important in the analysis of film cuts, given their fine temporal pattern within the timeline of the film. With ERPs, differences between cuts can be detected in a time resolved manner and potentially linked to specific stages of information processing (e.g., Reid and Striano, 2008; Sitnikova et al., 2008; Matran-Fernandez and Poli, 2015).

### 1.1. ERPs to shot change by cut: a brief review of findings

One of the first studies using ERPs to address edit cuts was conducted by Sitnikova and her research team (2008). In their study, ERPs following the moment of the cut were compared across three types of shot change: cuts in continuity with narrative coherence (take the bread loaf + cut a slice of bread), cuts that violated goal-related action requirements (take the bread loaf + place an iron on the loaf), and cuts that were unexpected but did not violate the goal-related requirements of the action (take the bread loaf + ironing a pair of the pants on an ironing board). Sitnikova et al. found significant differences in an anterior negativity N300/N400 component, which appeared in all three cases but was largest for unexpected cuts, and smallest for continuity cuts. In another study Reid and Striano (2008) focused on shot changes related to the completion of a previous activity in the scene. In their study, the shot after the cut could be the predictable completion of the previous action, or an unexpected ending. The ERPs after the unanticipated action endings showed a greater amplitude of the N400 component over frontal, central and parietal regions, with respect to anticipated cuts.

In a subsequent study, Francuz and Zabielska-Mendyk (2013) addressed the differences between related and unrelated cuts through ERP analysis. Unrelated cuts refer to those that involve a scene change (McKee, 1997), while related cuts refer to shot changes within the same scene. Related cuts maintain the visual unity of the filmic virtuality, at least within the immediate previous narrative context, whilst the unrelated cuts involve discontinuity (the concept of related and unrelated cuts was originally proposed in Carroll and Bever, 1976). Francuz and Zabielska-Mendyk found ERP differences in frontal electrode responses between 300 and 648 ms, unrelated cuts displaying more negative ERP compared to related cuts. This result is in line with the findings of Sitnikova et al. (2008) and Reid and Striano (2008). In addition, Francuz and Zabielska-Mendyk observed that the same difference extended over central electrodes (from 448 to 648 ms), and similar but opposed polarity differences in parietal electrodes, again with larger amplitude for unrelated cuts.

Heimann and her research team (2017) addressed the well-known 180-degree rule in film editing, typically used in dialogues between two characters. This is a conventional editing rule whereby all camera shots in the scene must be taken from one side of the virtual axis defined by an imaginary line linking the positions of the two interacting characters, and it is used to prevent confusion in the viewer (Murch, 1995/2001; Marimón, 2015). Heimann et al. found that cuts that violate the 180-degree rule triggered neuronal responses comparable to those that occur due to syntactic violations in language, such as an early left anterior negativity followed by a late positivity in the same area, and semantic violations such as a negative deflection around 400 ms in frontal scalp electrodes. This is in line with the studies discussed above addressing other breaches in filmic continuity.

In a different study, Matran-Fernandez and Poli (2015) compared ERPs elicited by shot changes (all consisting of related cuts) against a baseline condition without any cut. Compared to a no-cut baseline, shot changes produced a negative potential in the frontal electrodes, from around 100 ms up to 700 ms. Matran-Fernandez and Poli proposed to name the negative potential peaking between 380 and 420 ms, Post-Cut Negativity (PCN). This pattern, coherent with other studies (e.g., Reid and Striano, 2008; Sitnikova et al., 2008), defines the negative potential in frontal electrodes that is usually related with unexpected information, as is a typical neural reaction triggered by a cut. Analyzing the PCN they found a positive correlation with the duration of the shot preceding the cut, after controlling for luminance variations across cuts.

More recently, Andreu-Sánchez and her research team (2018) addressed the rhythmic aspect of editing techniques. To do this, they created two audio-visual clips with identical narrative content but different average shot length (ASL), a slow editing rhythm of 5.9 s and a faster one with an ASL of 2.4 s. The results showed that a faster edition triggered higher amplitude ERPs in occipital electrodes compared to slower editing, whilst slower editing triggered higher amplitude responses in the frontal and central scalp regions, compared to faster editing. Based on the results analyzed by ERP, frequency domain analysis and dipole estimation, they concluded that a faster editing rhythm increases attention, but at the same time decreases conscious processing.

Finally, Silva et al. (2019) analyzed editing cuts to study episodic memory encoding. They did not differentiate between types of cuts, but instead, they focused on differentiating those scenes that were remembered from those that had been forgotten, after viewing the film. They found significant differences in the ERPs of remembers vs. forgotten scenes between 600 and 1400 ms. Specifically, cuts that were recalled had elicited a more negative polarity in frontal, parietal and mid-temporal electrodes.

### 1.2. Inferences from the ERP findings so far

Based on the literature briefly reviewed above, the most characteristic response in the ERP signal triggered by shot changes is a large negative deflection in frontal electrodes between 300 and 700 ms, and a large positive deflection in the parietal scalp (Francuz and Zabielska-Mendyk, 2013). The negative deflection in frontal scalp electrodes could happen due to the overlap of evoked potentials with negative amplitudes, such as N300, N400 (Reid and Striano, 2008; Sitnikova et al., 2008) and SNW1 (Francuz and Zabielska-Mendyk, 2013), which can be associated to the neural processes triggered by unexpected events. This interpretation is supported by the fact that the amplitude of this frontal negativity is consistently larger when post-cut shots are least expected, such as for unrelated cuts (Francuz and Zabielska-Mendyk, 2013) and for violations of goal-related action expectations (Sitnikova et al., 2008). Sitnikova et al. (2008) noted that the latency of the N400 component for cuts is longer than in N400 arising from semantic inconsistencies when reading, due to the visual permanence of the incongruous information. Based on their investigations, they suggest that spatio-temporal information processing is reflected both in the N400 and in the late positivity.

In addition, the amplitude of the N400 could be related to the memory formation of the just-encoded event episode (Silva et al., 2019). The greater amplitude of N400 observed in the different comparisons between cuts reviewed above may reflect the mapping of visual input for semantic memory in a similar way that happens with language (Sitnikova et al., 2008). In particular, Matran-Fernandez and Poli (2015) suggest that this negative difference potential, located in the frontal and central scalp areas (which could be interpreted as N400 or more broadly as slow cortical potentials – SCP) may reflect the integration of new semantic information acquired after the shot change, built over the context of the previous shot.

On the other hand, the slow negative wave (SNW1) and slow positive wave (SPW) components, also present in some cases (Francuz and Zabielska-Mendyk, 2013) have been related to an orienting response (OR) or orienting reflex, reflecting an immediate response to a change in the environment (Sitnikova et al., 2008). The OR is a physiological and behavioral reaction that happens in response to new or unexpected stimuli (Öhman, 2021), preceding the orienting of sensory receptors toward salient events in the environment. Thus, the OR causes a non-conscious management of cognitive and attentional resources in order to process certain sensory information, called prominence or saliency (Evangelopoulos et al., 2013). Sitnikova et al. (2008) related the parietal positive deflection with the analysis of the observer content, even without the need for spatio-temporal inconsistencies to appear in the observed action. Regarding the positive deflection in parietal electrodes, Francuz and Zabielska-Mendyk (2013) ruled out the possibility that it reflects a P3 component, since the negative responses that co-occur in the frontal area make it incompatible with the P3a responses. Heimann and her research team (Heimann et al., 2017) detected a greater amplitude in the ERP between 400 and 600 ms after the cut (P4-6) in central right regions for shot changes that break the 180-degree rule, and lower in anterior left. This response, the authors interpreted, is associated with the adjustments of a detected violation without it reaching a level of visual awareness.

In summary, the consistent and large anterior negative and posterior positive deflections triggered in the ERPs have been generally related to the detection of incongruences due to the cut, and the ensuing adaptive responses in the brain. Here, the specifics vary from study to study, in part because of the differences in the concrete type of cut used, and the methodological and analytical approaches. For instance, one study showed that related cuts show late positive amplitudes in the central electrodes between 648 and 1800 ms, while unrelated cuts triggered negative amplitudes (Francuz and Zabielska-Mendyk, 2013), regardless of containing violations in goal-related action requirements (Sitnikova et al., 2008). In this sense, the responses triggered by unrelated cuts match with SNW2 (slow negative wave) responses, which usually appear after SNW1 responses (Francuz and Zabielska-Mendyk, 2013).

Despite most cut-related differences are seen in late components such as the ones discussed above, some studies have also reported earlier differences, though less consistently. Sitnikova and her research team (2008) reported the presence of components P1 (80 ms), N1 (180 ms) and P2 (220 ms), that reflect some variations due to the different types of shot changes, but not in all cases. They reported a greater negativity in the frontal and central areas, especially in the right hemisphere, between unrelated and related cuts as early as 150 to 250 ms. However, no differences were seen with expectation violations of goal-related action sequences. Heimann and her research team (2017) reported an early posterior negativity between 140 and 220 ms (N2), probably reflecting the recognition of a mismatch that implies bottom-up processes, followed by an early anterior positivity (P2). They interpret these early processes as the reflection that a reanalysis of the stimulus after a breach of expectation.

The differentiation between types of shot changes that has been studied with ERP (and other methods) has mainly focused on the coherence or incoherence of the narrative and visual content. Fewer studies, however, address variations between types of cuts used to create perceptual and narrative continuity, such as the present study, regarding continuity edits across shot scale and filming angle. The results of Andreu-Sánchez et al. (2018), larger frontal amplitude ERPs for cuts in slower compared to faster editing, and those by Matran-Fernandez and Poli (2015), who found larger Post-Cut Negativity after longer shots, fit well with this more fine-grained approach. Globally, these results would suggest that longer shots accumulate richer, more constraining pre-cut contexts, and therefore a more complex updating process would be required after the edit point (Matran-Fernandez and Poli, 2015).

### 1.3. Scope of the present research

Continuity editing is the basis for the construction of the narrative through the fragmentation of the staging, maintaining a sense of continuity in the viewer. Continuity is achieved via a set of editing techniques essential in film construction, conditioning how the spectator perceives the narrative (Reisz and Millar, 1971; Marimón, 2015), but their brain correlates have not been addressed before in detail. Instead, most studies have addressed the contrast between cut transitions preserving filmic (and narrative) continuity with cut transitions that break this continuity in different ways (scene transitions, semantic violations or breaches in the conventional rules). In the present study we address a comparison between different types of cut transitions meant to preserve continuity [that is, that they are related, in terms of time and space coherence, as described by Amiel, 2001/2005 and Burch, 1969], but change the scale or the filming angle across the cut. The possibility of locating differences in neural responses as a function of the shot scale and filming angle variations between the shots around the cut is consistent with the film theory developed in film editing. According to the scale law and the *30-*degree rule, the shot scale and angle variations larger than 30-degrees (45-degrees in North American film theory, e.g., Thompson, 1993) are relevant factors to produce a sense of continuity in the viewer, that is they are thought to go unnoticed (Marimón, 2015). One could therefore question whether variations of these, more subtle, types of edits will trigger differences in the typical neural responses to cuts, or any effects at all, in terms of ERPs, similar to the ones seen with more salient (and noticeable) cuts in the research reviewed above.

According to these past studies, the neuronal correlates registered from frontal and posterior electrodes are sensitive to different types of cuts between 300 and 700 ms (e.g., Sitnikova et al., 2008; Francuz and Zabielska-Mendyk, 2013). Therefore, if present, one would expect differences to be located mainly in the amplitudes of the signals between 300 and 700 ms, being negative in frontal electrodes and positive in posterior electrodes (Francuz and Zabielska-Mendyk, 2013). Specifically, according to previous research (Sitnikova et al., 2008), we expected to find differences in two time windows: from 250 to 350 ms, and from 350 to 600 ms.

## 2. Methods

**Figure 1:**
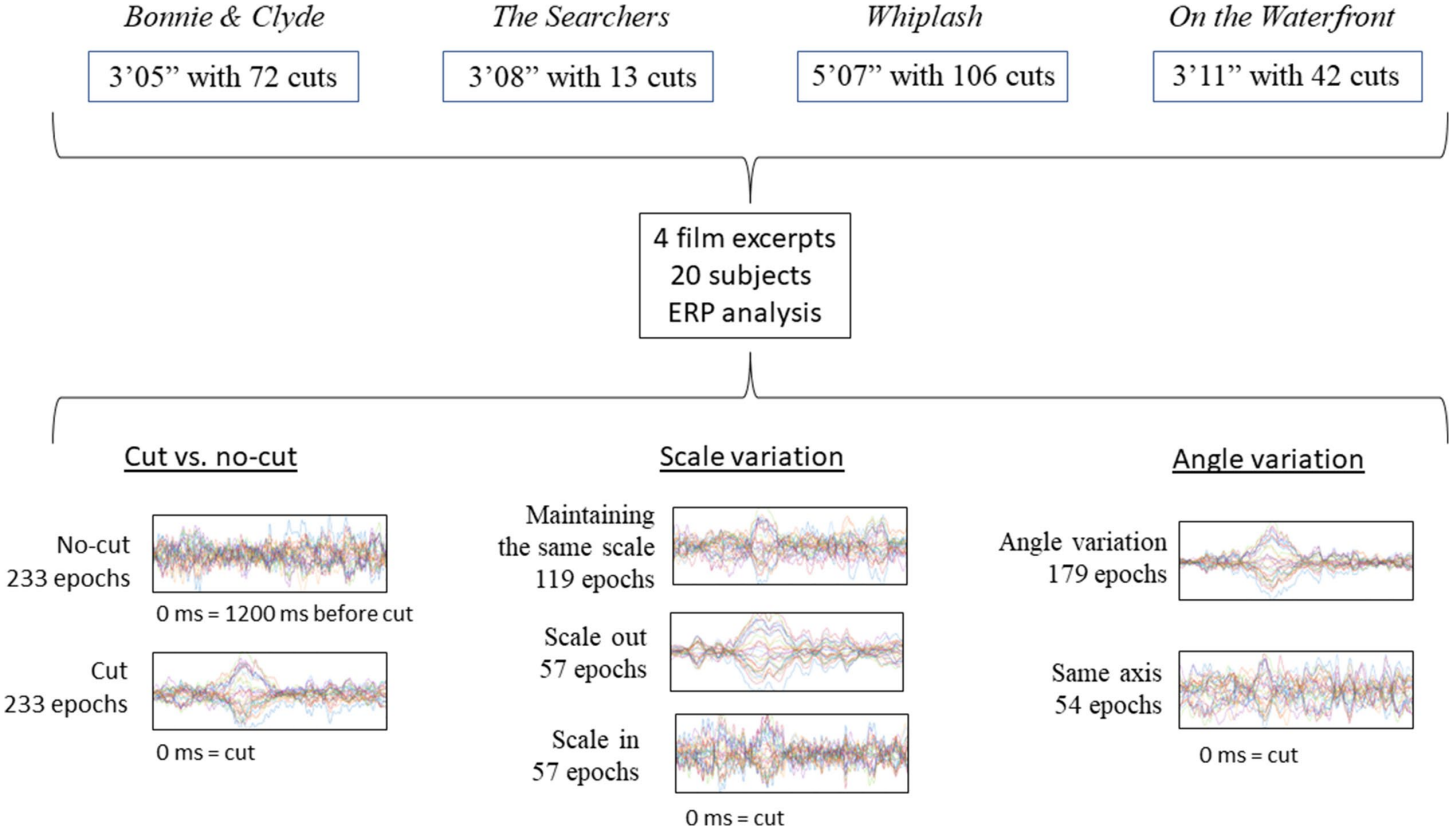
Visual representation of the study design.

These analyses have been performed on a dataset used in a previous research study (Sanz-Aznar et al., 2020, 2021), which addressed ERD/ERS but not ERPs. Below, we provide a brief description of the methods and design. The EEG recording was carried out on the participants while they passively viewed four film excerpts without any particular task. The analysis focused on the shot change (outgoing shot replaced by incoming shot) instant and subsequent time window. The specific pre-processing and data analyses are presented in full detail. Figure 1 visually represents the scheme of the experimental design.

### 2.1. Participants

Twenty subjects from the University of Aalborg, Denmark, chosen amongst undergraduate, master and, PhD students. The participants had an average age of 26 years (range 22–38 years old; 11 male and 9 female). Participants received 100 Danish Krone (approximately 13.42 Euro at the time of testing) worth of vouchers as compensation for their time, which they could redeem at a Danish cinema chain for tickets, popcorn and soft drinks. The experiment had the Aalborg University ethical approval signed letter with ID 2020-020-00504.

### 2.2. Materials and procedure

To carry out the experiment, we extracted four fragments from four feature films: *Bonnie & Clyde* (3′05″, Penn, 1967), *The Searchers* (3′08″, Ford, 1956), *Whiplash* (5′07″, Chazelle, 2014) and *On the Waterfront* (3′11″, Kazan, 1954). The indicated films can be classified within the institutional mode of representation (IMR) defined by Burch (1987/2006). This mode of representation encompasses the usual style of cinema consumed by the typical film spectator in western countries. The selected excerpts contained shot changes edited in continuity without temporal or spatial breaks, that conform to the definitions of absolute connection or articulated montage (Amiel, 2001/2005), as well as the categories of continuity or proximity in terms of spatiality and rigorously continuous or hiatus in terms of temporality (Burch, 1969). These categories defined by Amiel and Burch are the ones that allow the spectator to keep the virtual sensation of a continuous space–time, allowing the possibility of a continuity cut.

We chose 4 films with clear differences in terms of rhythm, esthetics and cinematographic technique, trying to compensate as much as possible the influence of spurious aspects of one specific film in the results (Table 1)^1^. For each film excerpt, we selected the cuts that fitted the continuity criteria mentioned above and, in addition, had incoming shots longer than 1000 ms (to ensure sufficiently long analysis epoch): 72 usable cuts in *Bonnie & Clyde* (14 not usable), 13 in *The Searchers* (1 not usable), 106 in *Whiplash* (6 not usable) and 42 in *On the Waterfront* (4 not usable). Usable cuts refer those continuity cuts in which the incoming shot is longer than 1000 ms, that suppose the analysis epochs. The average length of the shots for *Bonnie & Clyde* is 2.3 s (*SD* = 3.56, *Min* = 1.04, *Max* = 25.7), for *The Searchers* is 11.83 s (*SD* = 10.57, *Min* = 4.08, *Max* = 41.25), for *Whiplash* is 2.95 s (*SD* = 3.04, *Min* = 1.04, *Max* = 21.96) and for *On the Waterfront* is 4.26 s (*SD* = 2.37, *Min* = 1.33, *Max* = 13). To control for sequential effects, the order of the 4 excerpts was randomized for each subject. Before the viewing begins a white image with a central fixation cross was shown for 1′30″, and for 15″ between excerpts.

**Table 1:** Film excerpts characteristics.

Film	Excerpt timecode^a^	Color or B&W	Rhythmic ratio^b^	Narrative value^c^	Aesthetics^d^	Filmic style^e^
*Bonnie & Clyde*	01:46:48:01 to 01:49:53:02	Color	27.24	Inflection point. Strong conflict.	Modern	Transition
*The Searchers*	00:01:35:00 to 00:04:42:20	Color	4.79	Character presentation. No conflict.	Classical	Classical
*Whiplash*	00:23:33:13 to 00:28:40:13	Color	21.89	Inflection point. Strong conflict.	Modern	Post-classical
*On the Waterfront*	00:21:24:08 to 00:24:35:16	Black and White	14.45	Scene before an inflection point. Low conflict.	Classical	Classical

^a^Timecode format: Hour:Minute:Second:Frame. ^b^Average cuts per minute in the film excerpt. ^c^Narrative value in relation to the cinematographic narration and the conflict shown in the excerpt (McKee, 1997). ^d^Lighting style (Revault D’Allonnes, 1991). ^e^Cinematographic style (Bordwell, 1985; Langford, 2009; Thanouli, 2009).

**Figure 2:**
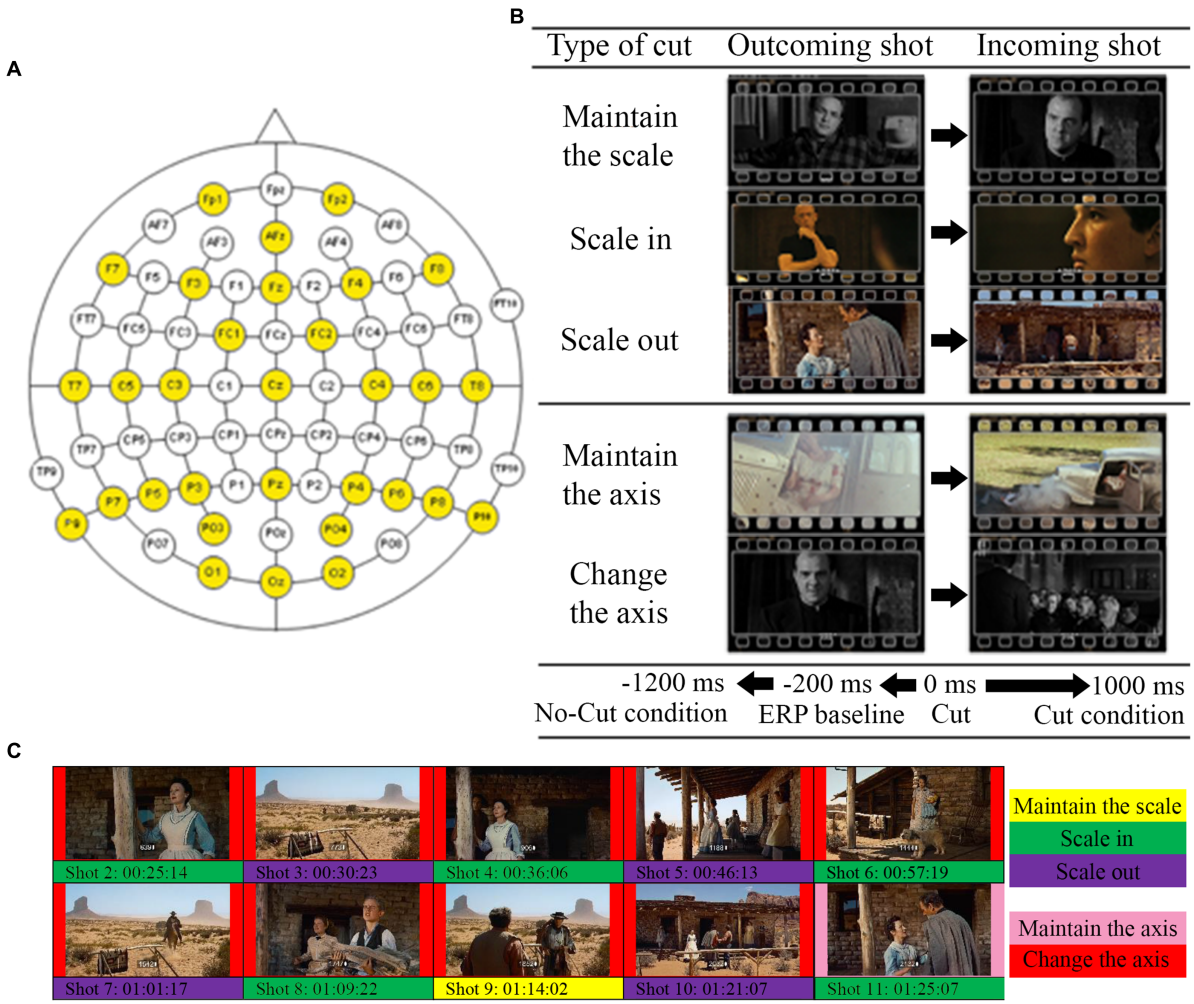
**(A)** Distribution of the electrodes used in the experiment (highlighted in yellow). **(B)** Examples for each type of cut used in the study. **(C)**. Example of type of cut segmentation for *The Searchers*.

The EEG recordings were carried out from 31 electrodes distributed according to the American Electroencephalographic Society 10–20 system, as the participants watched the movie excerpts without any particular task required (as movies are normally watched). The sampling frequency was 256 Hz. The devices used to amplify the EEG signal were two channel box g.Tec g.Gammabox connected to two biological amplifier g.Tec g.USB Amp. Both types of dispositive have 16 channels each, so connecting one device as a master and another as a slave allows 32 channels. The EEG signal was referenced to the right earlobe (online) and to the Fp1 electrode (offline). The recorded signal was analyzed using EEGLab (v2021.1) in MatLab (R2017a) environment, as well as the statistics. The recorded signal was re-referenced to the average of all scalp channels, Common Average Reference (Luck, 2014; Yao et al., 2019). The specific distribution of the electrodes on the scalp is represented in Figure 2A.

After recording the signal, a manual artifact rejection procedure was performed, and ICA was applied to separate the components caused by blinks and other muscle movements with a range of flagging from 90 to 100%. We applied a bandpass filter to the raw signal from 0.2 to 40 Hz. The average number of rejected epochs per subject was 18.22% (Min: 4.29%, Max: 42.06%). To carry out the ERP analysis, the signal was segmented in 1200 ms epochs, from −200 before to 1000 ms after the cut, with the 200 ms before to the cut used as baseline (following Matran-Fernandez and Poli, 2015).

### 2.3. Analyses

We selected two broad electrodes clusters of interest. The frontal cluster included Fp1, Fp2, Afz, F7, F3, Fz, F4, F8, Fc1 and Fc2, and the posterior cluster (parieto-occipital) included P7, P5, P3, Pz, P4, P6, P8, PO3, PO4, O1, Oz, O2, PO7 and PO8.

We first performed an analysis pooling all of the cuts together, simply comparing ERPs to cuts (stimulus) vs. no cut (absence of stimulus) segments (see below, for more details). The ERP for cut and no-cut condition are referenced to the same baseline, 200 ms before the cut [−200 ms, 0 ms], where 0 ms is the moment of the cut. Cut condition epochs included the first 1000 ms after the cut [0 ms, 1000 ms] and no-cut condition epoch included to the 1000 ms right before the reference [−1200 ms, −200 ms]. The analysis was run for comparison to the study by Matran-Fernandez and Poli (2015) comparing cuts vs. no-cuts between 380 and 420 ms using the Mann–Whitney test. As we re-referenced the electrodes to the average of all the scalp channels, we applied the analysis only to the frontal area and the posterior area independently, instead of grouping all the electrodes as done originally in Matran-Fernández and Poli.

Then we performed two comparative analyses contrasting the conditions (types of cuts) of interest (Figures 2B,C and Table 2). The first addressed scale variations, comparing the cuts as they scale in, scale out, or keep the same scale. To address cuts with scale variations we used a cluster-based permutation test, with the three conditions (keep, scale in, scale out) as independent variables. The second analysis focused on variations in filming angle, comparing cuts that keep the same filming axis (below 30°) with those that vary it (larger than 30°) using cluster-based permutation test. The statistical analysis Monte Carlo method, based on 500 randomizations (Heimann et al., 2017), with cluster correction, was applied in the two electrode clusters established (frontal and posterior) in both cases. We carried out time resolved point-by-point contrast across the first 0–1000 ms window after the cut.

**Table 2:** Number of usable cuts (epochs) per each condition.

Film excerpt	Cut	No-cut (before cut)	Keep scale	Scale in	Scale out	Same axis	Angle variation
*Bonnie & Clyde*	72	72	25	24	23	30	42
*The Searchers*	13	13	3	5	5	3	10
*Whiplash*	106	106	78	14	14	12	94
*On the Waterfront*	42	42	13	14	15	9	33
Total	233	233	119	57	57	54	179

Then, following Sitnikova et al. (2008), we focused on the average voltage amplitude in two time windows of interest; 250 to 350 ms, and 350 to 600 ms. These two time windows cover the typical times for the N300 and N400 epochs. Like Sitnikova, we used ANOVA to compare the different conditions (keep, scale in, scale out) as independent variables. We also applied *t*-tests for follow-up pairwise comparations. To reduce Type I error consequence of multiple comparison test *p*-values were adjusted for multiple comparison using Bonferroni correction and the accepted significance value for both cases was *p*-value ≤ 0.01. To estimate the effect size, we applied partial eta squared for ANOVAs and Cohen’s *d* for pairwise comparations.

## 3. Results

### 3.1. ERPs to cuts compared to no-cut periods

**Figure 3:**
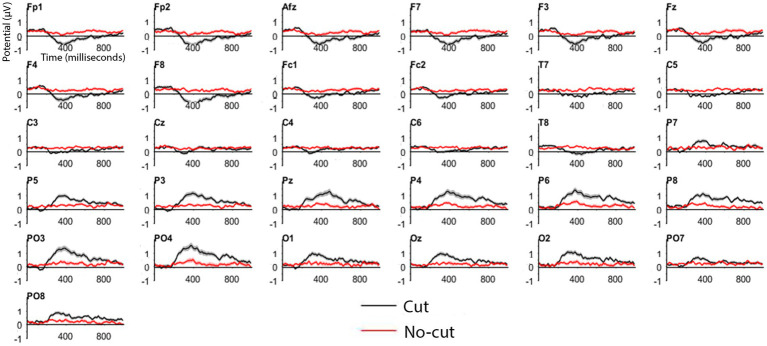
ERP for cut events (all cuts pooled, in black) and no-cut baseline (in red) for each electrode.

We first compared ERPs to cuts vs. a no cut baseline period of equal duration, as a reality check with respect to previous results using a similar method (e.g., Matran-Fernandez and Poli, 2015). We compared the average ERP in the interval [0–1000 ms] pooled over all the cuts (Figure 3, red line) with the average ERP of the interval [−1200 ms, −200 ms] prior to each cut (Figure 3, black line), which did not contain any cut. The ERPs were baseline corrected to the 200 ms interval before the cut [−200 ms, 0 ms]. As in previous experiments (e.g., Matran-Fernandez and Poli, 2015), the cut event triggered an ERP clearly differentiated from the signal where there is no shot change, as shown in Figure 3.

**Figure 4:**
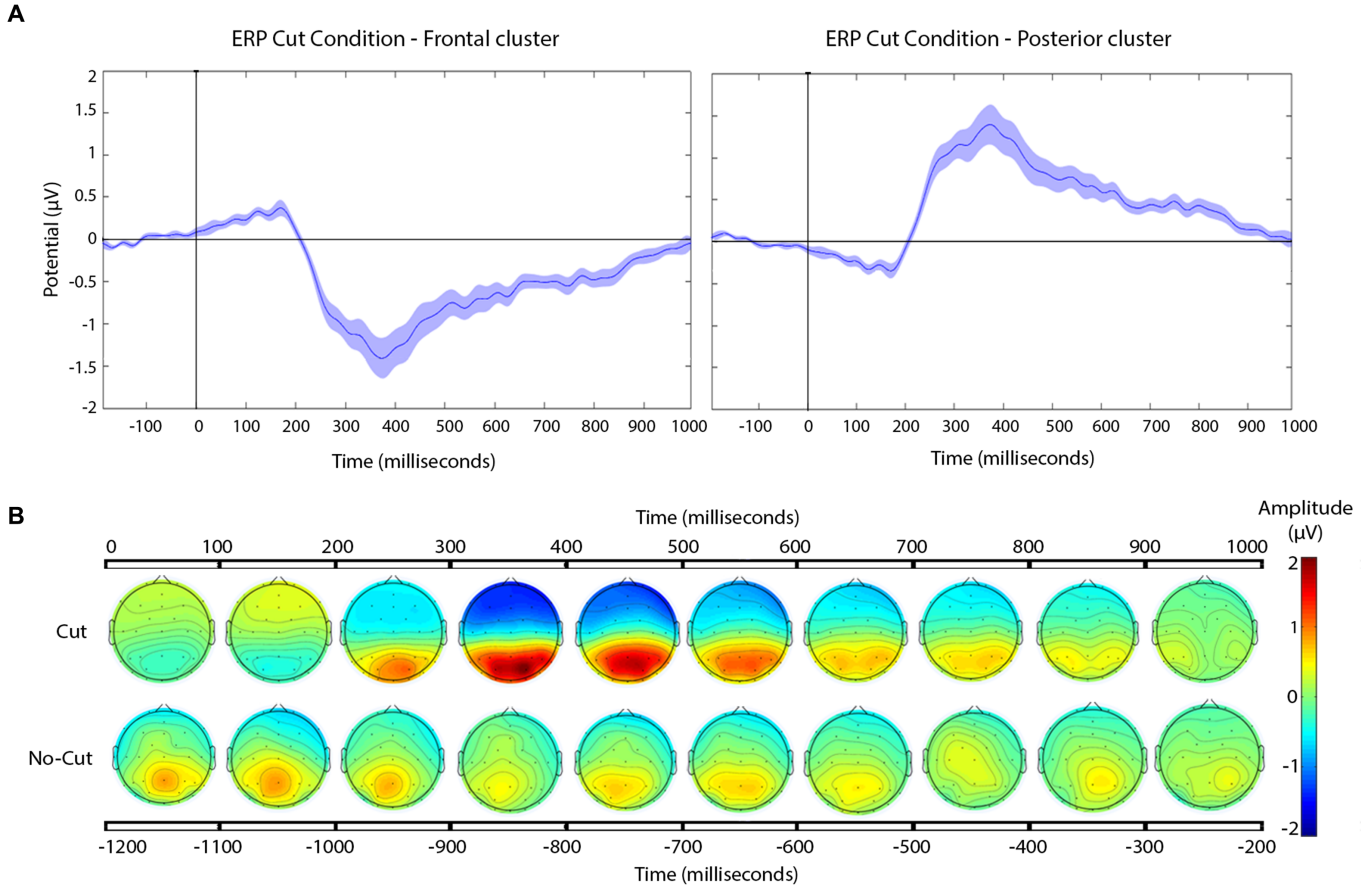
**(A)** Grand-average ERP for all cuts pooled for the frontal (left), and posterior (right) electrode clusters. Shaded areas represent the standard error of measurement (SEM) of ERP. **(B)** Scalp distribution of the voltage variation for all the cuts pooled together, compared to no cut, in 100 ms time steps.

Cut-evoked ERPs display a negative potential from 200 ms onwards in frontal electrodes, and a positive potential in the posterior areas, compared to no cut events. Figure 4A shows the cut condition for frontal and posterior electrodes.

The frontal cluster showed a clear negative potential peaking between 300 and 400 ms, followed by a progressive return to baseline until the end of the epoch. This posterior positive deflection peaks between 300 and 400 ms, followed by return to baseline until the end of the epoch. The scalp distributions (Figure 4B) of the average of the signal recorded following the cuts confirm the positive deflection in the posterior scalp, and a negative one in the frontal scalp, between 200 and 800 ms.

To further characterize this pattern, we applied a statistical approach similar to Matran-Fernandez and Poli (2015), comparing cut vs. no-cut between 380 and 420 ms using a Mann–Whitney test. We applied this comparison to the frontal and posterior electrode clusters separately, because unlike Matran-Fernandez and Poli, who used a whole scalp ERP (Global Field Power), our ERPs were already re-referenced the whole scalp.

We obtained a significant difference in both clusters, frontal (cut = −0.9261 μV vs. no cut = −0.043 μV *W* = 66, *p* < 0.01) as well as in posterior clusters (0.9229 μV vs. 0.0549 μV; *W* = 187, *p* < 0.01). Based on the results obtained in this across-the-board ERP analysis, we can differentiate cut condition from no-cut condition with our set of materials. We then concentrate on specific cut types separately for the frontal and the posterior clusters.

### 3.2. ERPs following scales changes across the cut

**Figure 5:**
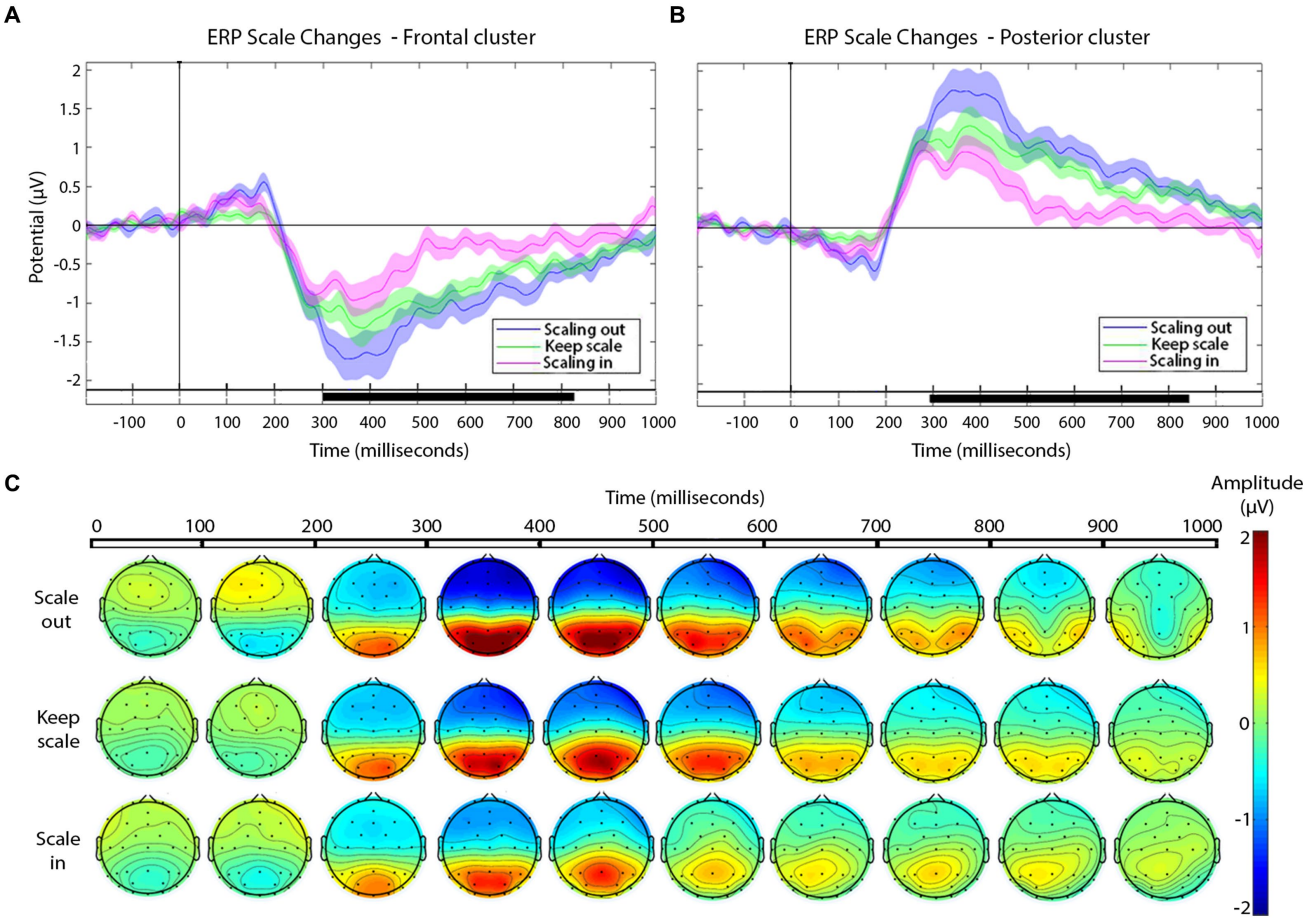
**(A,B)** Grand-average ERPs for each type of scale variation cuts (see legend) in the frontal **(A)** and posterior **(B)** electrode clusters. Shaded areas represent the SEM of ERP. In the timeline, black segments indicate significant effects in the cluster-based permutation test, *p*-value ≤ 0.01. **(C)** Scalp distribution of the negative and positive deflections, as a function of scale change across the cut, presented in windows of 100 ms after the cut.

To address scale variations between the shots across the cut, we carried out a point-by-point cluster-based permutation test (with one within-subject factor, scale change: scale in, scale out, or keep), separately for each electrode cluster (frontal, posterior), and established the significance level at *p*-value≤0.01. In frontal (Figure 5A) and posterior (Figure 5B) cluster analyses, the point-by-point cluster-based permutation test returned significant effects between 300 and 800 ms, according with the proposed hypothesis.

The magnitude of the negative frontal deflection (observed for all cuts pooled, in the previous analysis) varied as a function of the scale change across the edit (see Figures 5A,C). In particular, scale out produced a larger negative shift compared to those that keep the scale, whereas cuts with scale in produced a smaller negative shift, compared to keep-scale cuts. This was confirmed statistically, using the same time windows as in Sitnikova et al. The ANOVAs in the frontal cluster resulted significant in the 250–350 ms window [*F*(2,75) = 50.37, *p* < 0.01, *η^2^* = 0.573 (large effect)], and in the 350–600 ms window [*F*(2,189) = 171.59, *p* < 0.01, *η^2^* = 0.645 (large effect)]. The follow-up pair-wise *t*-tests confirmed the pattern described above in both time windows (ERP amplitude, scale out > maintain > scale in; see Table 3, for statistical values).

**Table 3:** *t*-test results for the two time windows (250–350 ms; 350–600 ms) and clusters (frontal and posterior) for paired comparations between scale in, scale out and maintain scale.

	Time window	Comparation	*df*	*t*	*P*	*d*	Effect size interpretation
Frontal	250–350 ms	Out vs. keep	19	−2.11	0.05	−0.473	Medium
		Out vs. In	19	−3.54	<0.01	−0.794	Medium
		In vs. Keep	19	−2.10	0.05	−0.469	Small
	350–600 ms	Out vs. keep	19	−1.60	0.125	0.359	Small
		Out vs. In	19	−4.06	<0.01	0.909	Large
		In vs. Keep	19	−7.33	<0.01	1.64	Trivial
Posterior	250–350 ms	Out vs. keep	19	2.85	0.01	0.638	Medium
		Out vs. In	19	3.85	<0.01	0.337	Medium
		In vs. Keep	19	2.05	0.05	−0.008	Trivial
	350–600 ms	Out vs. keep	19	3.35	<0.01	0.750	Medium
		Out vs. In	19	6.61	<0.01	1.479	Large
		In vs. Keep	19	6.91	<0.01	1.545	Large

In the posterior cluster (Figure 5B) the ANOVA returned significant effects around the window 300–800 ms. The posterior electrode cluster displayed a positive shift between 300 and 800 ms, consistent with what had been observed in the overall analyses (all cuts pooled). Like for the negative shift in frontal electrodes, the amplitude of the posterior positive shift depended on the type of scale change across the cut, following a similar pattern: with respect to keeping scale, scale out produced stronger shift, and scale in produced a decrease in the positive shift. For consistency, we used the same time windows as before for the statistical confirmation of this pattern. The ANOVAs for the window 250–350 ms and the 350–600 ms window in the posterior were both significant {respectively, [*F*(2,75) = 55.95, *p* < 0.01, *η^2^* = 0.599 (large effect)], [*F*(2,189) = 45.03, *p* < 0.01, *η^2^* = 0.605 (large effect)]}. The follow up paired *t*-tests confirmed the pattern with the significance levels (see Table 3).

### 3.3. ERPs following angle variations across the cut

**Figure 6:**
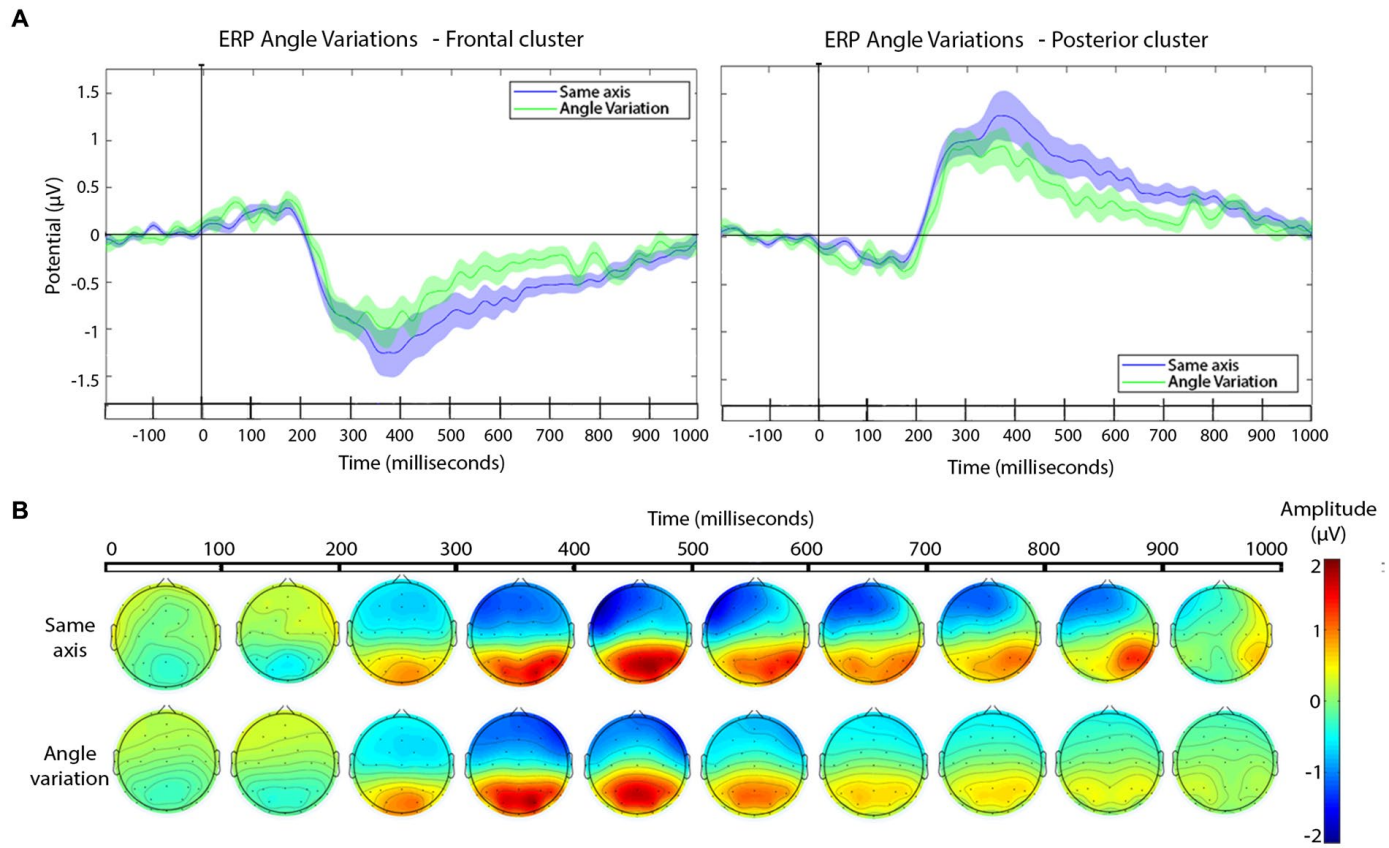
**(A)** ERPs for edits with and without angle variation across the cut for the frontal (left) and the posterior (right) electrode clusters. Shaded areas represent the SEM of ERP. In the timeline, significant effects in the cluster-based permutation test, *p*-value ≤ 0.01 are indicated in black (no significant effects were found). **(B)** Scalp distribution of the voltage variations as a function of the angle variation (same or different axis) across the cut, presented in windows of 100 ms after the cut.

To address shot changes that vary the filming axis we started with a point-by-point approach as before, using cluster-based permutation test. The outcome is not as conclusive as for scale variations (see, Figure 6A).

As can be seen in Figure 6A, there were no significant differences in the ERP between 200 and 800 ms, the approximate segment of interest. For completeness, the scalp distributions are shown in Figure 6B. Therefore, the ERPs to this type of cut followed the general profile that was expected from previous research, as well as our own analysis of the pooled dataset, and the scale variation cuts: frontal negative deflection and posterior positive deflection, over the 200–800 ms window, with peaks happening 300–400 ms. Yet, there were no detectable ERP changes as a function of filming angle variation across the cut. The *t*-test for the window 250–350 ms and the 350–600 ms window in the frontal and posterior cluster were no significant. Results can be consulted in Table 4.

**Table 4:** *t*-test results for the two time windows (250–350 ms; 350–600 ms) and clusters (frontal and posterior) for paired comparations between same or different axis.

	Time window	*df*	*t*	*P*	*d*	Effect size interpretation
Frontal	250–350 ms	19	−0.62	0.54	−0.139	Trivial
	350–600 ms	19	−2.40	0.03	−0.536	Medium
Posterior	250–350 ms	19	−1.08	0.29	0.241	Small
	350–600 ms	19	2.78	0.01	0.622	Medium

## 4. Discussion

At variance from most ERP studies addressing continuity violations or unrelated filmic cuts, we addressed potential differences between different types of continuity edits. Understanding how the human brain captures shot changes across cuts is important because they are amongst the most widely used devices for film composition in cinematography. In particular, we set out to analyze the ERPs following related film edits which involved differences in shot scale (scale in, scale out, or keep scale) and in shot angle (angle variation, vs. same axis). For example, scale and angle variation across shots help articulate different space and unity pieces and adding a hierarchical value of different shots within the scene whilst maintaining a feel of continuity in the viewer (Reisz and Millar, 1971; Marimón, 2015).

In order to align our results with those of previous studies we first analyzed the ERPs to all cuts pooled together, with respect to a no-cut baseline. Please note that such reality check does not only seek confirmation of previous findings, but also helps bring some coherence across the very different filmic materials and potential viewing conditions across different studies. Our results were overall consistent with the previous literature that analyzed the ERP triggered by shot changes, with very clear ERPs evoked by editing cuts (Francuz and Zabielska-Mendyk, 2013; Andreu-Sánchez et al., 2018; Andreu-Sánchez and Martín-Pascual, 2021). However, because of methodological difference we can only confirm the existence of the PCN (380–420 ms), described by Matran-Fernandez and Poli (2015) for all the electrodes, in frontal electrodes. Based on our across-the-board ERP analysis, we saw a negative potential appearing at a frontal cluster and a positive posterior deflection, peaking between 300 and 400 ms, both followed by return to baseline until the end of the epoch. This result coincides in frontal electrodes with that obtained by Matran-Fernandez and Poli, whose working hypothesis was that the cut would trigger an N400, due to abrupt change of visual information. However, our results differ in the posterior cluster, which in our case shows a clear positive deflection.

This divergence between results could be related to the different references used to calculate the ERPs across the two studies (averaged earlobes in Matran-Fernandez and Poli, 2015; whole scalp average in ours), making the direction and distribution of electrical fields not direcly comparable between studies. However, the temporal profile of the frontal negativity seen in our results fits well with the timing in Matran-Fernandez & Poli, as well as with that of several other ERP studies to filmic cuts (Reid and Striano, 2008; Sitnikova et al., 2008). About the positive deflection detected in posterior cluster, our results are generally coincident with most previous research (e.g., Francuz and Zabielska-Mendyk, 2013; Andreu-Sánchez et al., 2018; Andreu-Sánchez and Martín-Pascual, 2021).

In general, the ERP pattern can be described as a negative component in frontal electrodes and positive deflection in posterior electrodes, extending roughly from 200 to 800 ms, with peaks between 200 and 400 ms. These results, coincide with abundant previous literature (Reid and Striano, 2008; Sitnikova et al., 2008; Francuz and Zabielska-Mendyk, 2013), and provide grounds for the investigation of variations as a function of cut type in the time windows of interest in the present study. These neuronal correlates have been related to the processing of cognitive inconsistencies triggered by the sudden change of visual input produced across the editing cut (Silva et al., 2019). This interpretation is coherent with our own results, but also with other studies addressing other ERP components, such as the study by Calbi et al. (2017). Calbi et al. analyzed the Kuleshov effect (Kuleshov, 1934/1994) by focusing on the ERP N170, which is characteristically elicited by faces. They concluded that the Kuleshov effect is the consequence of an attribution of expectations set by the shot preceding the cut as a function of the emotional coherence, or incoherence, across the edit.

As the main focus of our research, we registered effects in this frontal-negative and posterior-positive deflections related to different types of cuts depending on the scale variation. The negative-going deflection in the frontal cluster had a graded amplitude depending on the type of scale change across the cut, with similar scalp distributions in all cases: The largest shift corresponded to scale out, whilst scale in cuts led to the smallest (yet still significant) deflection, with ERPs to cuts keeping the scale falling in-between. Given the timing and scalp distribution of these negative shifts, one could relate them to the N300 and N400 components, following the interpretation of earlier ERP studies addressing film cuts (Hamm et al., 2002; Kumar et al., 2021). In particular, amplitude differences in the N300 and N400 are common in research that compares different types of cuts (Reid and Striano, 2008; Sitnikova et al., 2008), although they have so far been studied for edit transitions involving some degree of narrative or filmic continuity breach such as related vs. unrelated, predictable vs. unpredicted action continuations, or semantic and compositional incongruences. For instance, larger N400s are usually seen in cuts with action incoherence (Reid and Striano, 2008; Sitnikova et al., 2008). Based on these interpretations and the effects observed here, we might suggest the hypothesis that scale reductions from one shot to the next (scale in) may reduce the perception of incoherence, compared to keeping scale, and to a larger extent, scaling out. According to Matran-Fernandez and Poli (2015), the amplitude variation in N400 may reflect the integration of new semantic information built on the context of the previous shot. Interpreting our results under this light, a reduction in shot scale may imply a smaller amount of new information to integrate than scaling out.

On the other hand, the positive shift in the posterior cluster between 300 and 800 ms also varied in amplitude as a function of cut type in our results. Again, and in parallel to the anterior cluster, scaling out produced the largest amplitude in this component, followed by keep and then by scaling in. According to prior ERP studies on film editing, amplitude variations in the posterior responses between 350 and 450 ms have been related to a greater inconsistency between shots across an edit (Francuz and Zabielska-Mendyk, 2013). Based on these previous investigations, scale out would enhance neural responses related to visual incongruity, with these effects being smoother in the case of scale in.

Overall, the results from the frontal and parieto-occipital clusters are coherent, allowing us to propose the interpretation that scale out produce greater incoherence (at least, in terms of neural correlates) than scale in, with cuts that keep the scale constant being an intermediate case. According to this interpretation, one would therefore expect that scale out would be more noticeable on average to the viewers than scale in across shots in a related cut. This relationship between scale in/out and visibility is known in professional film editing and well documented in film editing handbooks. Specifically, to achieve smooth flow in a scene, the editing should be designed from the most open shots to closed ones (Reisz and Millar, 1971). Since it is not possible to sustain an incremental reduction of scale throughout each cut along a scene, the correspondence law (Marimón, 2015) is often applied. The correspondence law consists of keeping the same scale (among other aspects) to keep editing flow. The ERP results thus coincide with the praxis in cinema montage. However, it would be interesting to test these predictions in a behavioral experiment, using perhaps a similar approach as that of Smith and Henderson (2008) or Magliano and Zacks (2011), where they asked participants to explicitly detect edit points whilst watching a film.

Regarding the other main focus of our study, cuts based on the angle variation, the results did not show significant differences in the ERPs. In this case we cannot confirm the expectation, according to the film theory. According to the *30-*degree rule (Marimón, 2015) the cuts that imply less than 30^0^ of variation (including those with no angle variation) should produce less continuity feeling in the viewer than the cuts that change the camera angle by more than 30^0^. Cuts that break the *30-*degree rule are thought to be more ‘aggressive’ from a cognitive point of view and perceived by the viewer as a jump in the continuity flow (Königsberg, 1987; Marimón, 2015). Please note that although we have not found significant results in this case, there could be an actual difference, but the effect size might be small and our measurement not sensitive enough. Perhaps related to this, Smith et al. (2012) proposed that spatial memory is worse than identification memory, and therefore the spectator does not retain an allocentric representation of scenes. Maybe this poorer spatial reference explains the absence of significant results in N300 and N400 for angle variations. If this speculation where true, and in the light of our results, one would expect that, overall, angle variations should be less noticeable consciously than variations in scale.

In another investigation using the same dataset used here some of us have addressed (Sanz-Aznar et al., 2023) similar comparisons between cuts through ERD/ERS analysis, instead of ERPs. This analyses found differences between related cuts that vary shot angle vs. those that keep the same axis, as well as cuts that vary the scale of the shot vs. those that keep it. These differences were found in the first 125 ms after the cut, in central and posterior electrodes and between 300 and 1000 ms in frontal and parietal electrodes, in the frequency range Theta (3–7 Hz) and Delta (0.5–3 Hz). Theta frequency results relevant in the first 400 ms and delta from 500 ms.

## 5. Conclusion

We have investigated ERPs to film cuts using widely accepted editing techniques employed to confer a sense of continuity in the viewer, such as scale in/out, and angle variation. In all cases, these techniques have been developed with intuition and practice by filmmakers and montage professionals, to maintain a sense of continuity and flow across cuts. Our results seem to provide grounds for validation of this common editing practice from a neuroscience perspective and, importantly, may lay the basis for addressing other common editing techniques or even test new editing variations in a principled way. As one potential limitation of the current experimental approach, it should be noted that the subject’s perceptive evaluation of the cut events are not taken into account. This decision was made consciously, because we sought to reproduce the cinematographic viewing experience as much as possible, hence favoring a passive viewing without specific task. Finally, the present results also raise some expectations regarding the viewer’s awareness of visual edits in films. The results obtained allow us to hypothesize that the spectator could have a greater awareness of the visual transitions involving scaling out than when scaling in, with shot changes that keep constant scale as an intermediate typology. This means that in film editing, when a cut scales in, it should be more invisible for the spectator than a cut that scales out. No significant differences have been detected when comparing cuts with different camera angles between shots, and so this case remains less conclusive.

## Data availability statement

The datasets presented in this study can be found in online repositories. The names of the repository/repositories and accession number(s) can be found at: https://osf.io/m97xf/?view_only=a0427e4b8cee4c51bb09b0f978b95883.

## Ethics statement

The studies involving human participants were reviewed and approved by the experiment had the Aalborg University ethical approval signed letter with ID 2020-020-00504. The patients/participants provided their written informed consent to participate in this study.

## Author contributions

JS-A and LB experimental design and carried out experiments. SS-F and JS-A performed data analysis and wrote the article. All authors contributed to the article and approved the submitted version.

## Funding

This research was supported by the Ministerio de Ciencia e Innovación (PID2019-108531GB-I00 AEI/FEDER), AGAUR Generalitat de Catalunya (2021 SGR 00911) grants to SS-F and NextGenerationEU (Margarita Salas) grant to JS-A.

## Conflict of interest

The authors declare that the research was conducted in the absence of any commercial or financial relationships that could be construed as a potential conflict of interest.

## Publisher’s note

All claims expressed in this article are solely those of the authors and do not necessarily represent those of their affiliated organizations, or those of the publisher, the editors and the reviewers. Any product that may be evaluated in this article, or claim that may be made by its manufacturer, is not guaranteed or endorsed by the publisher.

## Footnotes

^1^To define the technical and esthetic differences between the film excerpts, we resorted to analysis references that provide general classifications regarding lighting esthetics (Revault D’Allonnes, 1991), film style (Bordwell, 1985; Langford, 2009; Thanouli, 2009), narrative-structural description (McKee, 1997), rhythmic ratio or cutting rates (Redfern, 2022) and the technical characteristic of whether the excerpt is black and white or color. Taking these characteristics as a reference, a first list of different fragments extracted from 20 films was elaborated. This first selection was reviewed by two experts in film analysis (Juan José Caballero Molina y Daniel Jariod Dato). Based on the report prepared by the experts, the list was definitely reduced to the four selected fragments.
